# Lung inflammation induced by silica particles triggers hippocampal inflammation, synapse damage and memory impairment in mice

**DOI:** 10.1186/s12974-022-02662-0

**Published:** 2022-12-16

**Authors:** Patrick R. Suman, Lisiane S. Souza, Grasielle C. Kincheski, Helen M. Melo, Mariana N. Machado, Giovanna M. C. Carvalho, Fernanda G. De Felice, Walter A. Zin, Sergio T. Ferreira

**Affiliations:** 1 Institute of Biophysics Carlos Chagas Filho, Universidade Federal do Rio de Janeiro, Rio de Janeiro, Brazil; 2 Institute of Medical Biochemistry Leopoldo de Meis, Universidade Federal do Rio de Janeiro, Rio de Janeiro, Brazil; 3 Pedro Ernesto University Hospital, Universidade do Estado do Rio de Janeiro, Rio de Janeiro, Brazil; 4 D’Or Institute for Research and Education, Rio de Janeiro, Brazil; 5 Centre for Neuroscience Studies, Department of Biomedical and Molecular Sciences & Department of Psychiatry, Queen’s University, Kingston, Canada

**Keywords:** Silicosis, Lung inflammation, Brain inflammation, Synapse damage, Cognitive impairment, Dementia

## Abstract

**Background:**

Considerable evidence indicates that a signaling crosstalk between the brain and periphery plays important roles in neurological disorders, and that both acute and chronic peripheral inflammation can produce brain changes leading to cognitive impairments. Recent clinical and epidemiological studies have revealed an increased risk of cognitive impairment and dementia in individuals with impaired pulmonary function. However, the mechanistic underpinnings of this association remain unknown. Exposure to SiO_2_ (silica) particles triggers lung inflammation, including infiltration by peripheral immune cells and upregulation of pro-inflammatory cytokines. We here utilized a mouse model of lung silicosis to investigate the crosstalk between lung inflammation and memory.

**Methods:**

Silicosis was induced by intratracheal administration of a single dose of 2.5 mg SiO_2_/kg in mice_._ Molecular and behavioral measurements were conducted 24 h and 15 days after silica administration. Lung and hippocampal inflammation were investigated by histological analysis and by determination of pro-inflammatory cytokines. Hippocampal synapse damage, amyloid-β (Aβ) peptide content and phosphorylation of Akt, a proxy of hippocampal insulin signaling, were investigated by Western blotting and ELISA. Memory was assessed using the open field and novel object recognition tests.

**Results:**

Administration of silica induced alveolar collapse, lung infiltration by polymorphonuclear (PMN) cells, and increased lung pro-inflammatory cytokines. Lung inflammation was followed by upregulation of hippocampal pro-inflammatory cytokines, synapse damage, accumulation of the Aβ peptide, and memory impairment in mice.

**Conclusion:**

The current study identified a crosstalk between lung and brain inflammatory responses leading to hippocampal synapse damage and memory impairment after exposure to a single low dose of silica in mice.

## Introduction

Mounting evidence indicates that a crosstalk between peripheral and central inflammation can lead to brain dysfunction and neurodegeneration [1–3]. For example, chronic kidney disease has been associated with cognitive impairment, delirium, encephalopathy, and dementia [4, 5]. Type 2 diabetes and obesity, characterized by peripheral inflammation and insulin resistance, are risk factors for dementia [6–8] and for major depressive disorder [9]. Gut microbiota has also been implicated in brain–periphery crosstalk [10]. Exposure to microbial amyloids in the gastrointestinal tract can accelerate alpha-synuclein aggregation in the gut and brain, and lead to enhanced microgliosis and astrogliosis, suggesting that bacterial amyloid may function as a trigger to initiate brain inflammation and alpha-synuclein aggregation in synucleinopathies [11, 12].

Robust evidence further indicates that acute peripheral inflammatory conditions, including viral and bacterial infections, can trigger brain inflammation and dysfunction, resulting in cognitive decline or neuropsychiatric conditions [13, 14]. For example, recent studies demonstrate that systemic inflammation induced by SARS-CoV-2, the etiologic agent of COVID-19, activates brain toll-like receptors (TLRs) and upregulates brain tumor necrosis factor-α (TNF-α) and interleukin-6 (IL-6) signaling, triggering synapse damage and leading to depressive and cognitive symptoms in COVID-19 patients [15]. Collectively, multiple lines of evidence indicate that both acute and chronic, low grade peripheral inflammation can trigger brain inflammation and progressively lead to brain dysfunction underlying cognitive decline and dementia [13–17].

Silicosis is a major occupational lung disease, with a significant impact on health systems and on the quality of life of workers in many industries [18, 19]. Exposure to silica (SiO_2_) particles induces chronic lung inflammation, including immune cell infiltration, macrophage activation and release of pro-inflammatory cytokines, e.g., TNF-α, interleukin-1β (IL-1β) and IL-6, resulting in tissue fibrosis, alveolar collapse and lung dysfunction [19–21].

Clinical and epidemiological evidence points to an association between pulmonary function and cognitive impairment. A recent meta-analysis of longitudinal studies of individuals with impaired pulmonary function found an increased risk of dementia in such individuals [22], and this appears more pronounced for restrictive pulmonary impairment than for obstructive lung disease [23]. Recent studies indicate that both neurodegeneration and vascular brain lesions may underlie the association between pulmonary dysfunction, memory impairments and dementia [24, 25]. However, the mechanistic underpinnings of the connection between lung and brain dysfunction remain unclear.

Here, we investigated the crosstalk between lung inflammation, brain inflammation and memory in a mouse model of silicosis. We report that lung inflammation in mice exposed to silica particles is accompanied by hippocampal inflammation, synapse damage and memory impairment.

## Methods

### Animals

Experiments were performed on 8- to 10-week-old male Swiss mice. Animals were housed in groups of five per cage with free access to food and water, under a 12-h light/dark cycle with controlled room temperature (21 ± 2 °C). Mice were randomly divided into two groups. In the control group (Ctrl), mice received an intratracheal (i.t.) administration of 0.05 mL sterile saline solution (0.9% NaCl). Silica-exposed animals (Si) received an intratracheal administration of 2.5 mg silica (SiO_2_; particle size: 500 nm–10 μm, 80% of the particles between 1 and 5 μm; S5631, Sigma Chemical Co., St. Louis, USA) suspended in 0.05 mL saline, as previously described in murine models of acute silicosis [26, 27]. Animal behavior was analyzed 24 h or 15 days after saline or silica administration. This study was approved by the Ethics Committee on the Use of Animals, Health Sciences Center, Federal University of Rio de Janeiro.

### Tissue collection and preparation

Mice were anesthetized with 1.5 ml/kg of a solution containing 10% ketamine and 2% xylazine immediately after behavioral tests. Bilateral hippocampi and lungs of Ctrl and Si animals were collected, immediately frozen in liquid nitrogen, and stored at − 80 °C until use in Western blotting or ELISA assays.

### Lung histology

Histology was performed as previously described [20, 26]. Morphometric analysis was performed in granuloma-free tissue areas using an integrating eyepiece with a coherent system of a 100-point and 50 lines (known length) grid coupled to a conventional light microscope (Axioplan; Zeiss). Analysis was performed in 10 random, non-overlapping fields with 200× magnification.

For cellularity analysis, total numbers of mononuclear (MN) and polymorphonuclear (PMN) cells in granuloma-free lung tissue areas were counted in each animal across 10 random non-overlapping microscopic fields at 1000× magnification. Data are presented as cell count percentages and cell numbers/tissue area.

### Hippocampal and lung cytokines

Hippocampi and lungs were homogenized in ice-cold PBS with protease and phosphatase inhibitors (Pierce–Thermo Scientific, Rockford, IL), and were centrifuged for 10 min at 26,500×*g* at 4 °C. Supernatants were collected and assayed in duplicate by ELISA for the following cytokines: Interleukin-1β (IL-1β, Thermo Scientific, Rockford, IL), TNF-α (Biolegend, San Diego, CA), interleukin-6 (IL-6; R&D Systems, Minneapolis, MN). Amyloid-β42 was quantified using a mouse Aβ ELISA kit (Invitrogen/Thermo, cat # KMB3441).

### Western immunoblotting

Samples were thawed and homogenized in 25 mM Tris–HCl, pH 7.5, 150 mM NaCl, 1% NP‐40 (Invitrogen), 1% sodium deoxycholate, 0.1% SDS, 5 mM EDTA, 1% Triton X‐100 and phosphatase and protease inhibitor cocktail. Protein concentrations were determined using the BCA kit (Pierce-Thermo Scientific, Rockford, IL). Samples containing 30 µg protein were resolved in 4–20% polyacrylamide Tris–glycine gels (Novex; Invitrogen, Grand Island, NY) and transferred to nitrocellulose membranes at 300 mA for 1 h. Blots were incubated with Odyssey^®^ blocking buffer (Li-Cor; Lincoln, NE) at room temperature for 1 h and were incubated with primary antibody diluted in blocking buffer at 4 °C overnight. Primary antibodies used were anti-PSD-95 (1:1000; Santa Cruz, Dallas, TX), anti-synaptophysin (1:1000; Sigma, St Louis, MO), anti-β-actin (1:15,000; Abcam, Cambridge, UK), anti-cyclophilin (1:10,000; Abcam, Cambridge, UK), anti-amyloid precursor protein (APP) (1:1000 Zymed- Thermo Scientific, Rockford, IL), anti-Bace1 (1:2,000; Milliporesigma, Burlington, MA #5940), and anti-p-AKT-Ser473 (1:1500; Cell Signaling, Danvers, MA). Membranes were incubated with IRDye-conjugated secondary antibodies (1:10,000; LI-COR Biosciences, Lincoln, NE) at room temperature for 1 h, imaged on an Odyssey Imaging System (LI-COR Biosciences, Lincoln, NE), and analyzed using NIH Image J.

### Behavioral tests

#### Open field

Mice were placed at the center of an open field arena (30 cm × 30 cm × 45 cm) for habituation, and their activity was recorded for five minutes. Total distance traveled and time spent in the central square were automatically quantified using Any-maze® video-tracking system (Stoelting Inc., Kiel, WI). The arena was cleaned with 20% ethanol between trials to eliminate olfactory cues.

#### Novel object recognition test (NOR)

The novel object recognition test was performed in the open field arena with objects fixed to the box using tape. The test was video recorded for behavior readout [28]. During training and test sessions, animals were placed at the center of the arena, and exploratory behavior toward both objects was recorded for 5 min. The arena was cleaned with 20% ethanol between trials to eliminate olfactory cues. The training session was performed in the presence of two identical objects. One of the two objects used in the training session was then replaced by a novel object for the test session, carried out one and a half hours after training. Sniffing and touching the object were considered exploratory behavior, and the amount of time spent exploring each object was registered by a trained researcher [28].

### Statistical analyses

All datasets were submitted to the Shapiro–Wilk normality test. Specific statistical tests employed are mentioned in figure legends. Datasets showing normal distribution were analyzed by ANOVA or Student’s *t-*test. Histological datasets were analyzed by ANOVA and differences were considered statistically significant with *p* < 0.05. Data from the NOR task were analyzed using a one-sample Student’s *t*-test, comparing the exploration time of the novel object to the fixed value of 50% (chance level) as previously described [28–31]. All analyses were performed using GraphPad Prism 8 (GraphPad Software; La Jolla, CA). Results are expressed as means ± SEM, and corresponding t-values.

## Results

### Intratracheal administration of silica induces alveolar collapse and lung infiltration by polymorphonuclear (PMN) cells

**Fig. 1:**
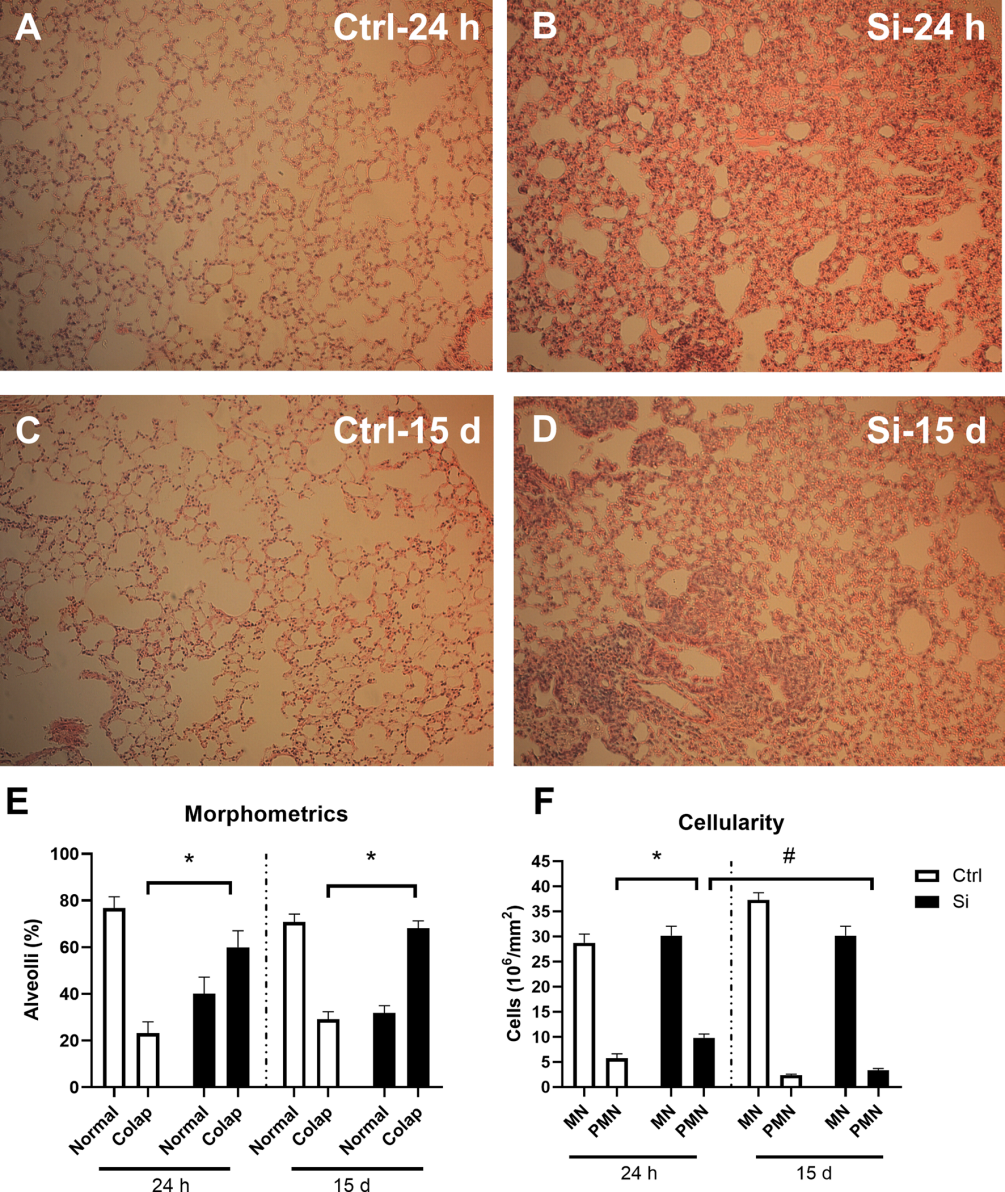
Alveolar collapse and lung infiltration by PMN cells in silica-instilled mice. Representative photomicrographs of lungs from control (Ctrl) or silica-instilled (Si) animals. **A** Ctrl 24 h; **B** Si 24 h; **C** Ctrl 15 d; **D** Si 15 d. **E** Percentage of collapsed alveoli in Ctrl (white bars) or Si (black bars) animals. **F** Cellularity expressed as total cell counts per area. Data are expressed as mean ± SEM; white bars represent Ctrl mice, and black bars represent silica-instilled (Si) mice. Two-way ANOVA with Duncan post hoc test. *p < 0.05 between Ctrl and Si; ^#^*p* < 0.05 between 24 h and 15 d. *N* = 7–10 mice per experimental group

Lung histology showed that intratracheal administration of SiO_2_ (silica) particles caused a significant increase in the percentage of collapsed alveoli compared to control, saline-instilled mice (F (3, 25) = 22.2908, *p* = 0.000003), both 24 h (*p* = 0.00007) and 15 d (*p* = 0.00007) after administration of silica (Fig. 1A–E). The number of polymorphonuclear (PMN) cells in the lungs was significantly increased 24 h after administration of silica (F (6, 48) = 13.452, *p* = 0.000166), and returned to baseline values 15 d after administration (*p* = 0.000066). No differences were observed in lung mononuclear cell numbers in silica-treated mice.

### Intratracheal administration of silica induces lung inflammation

**Fig. 2:**
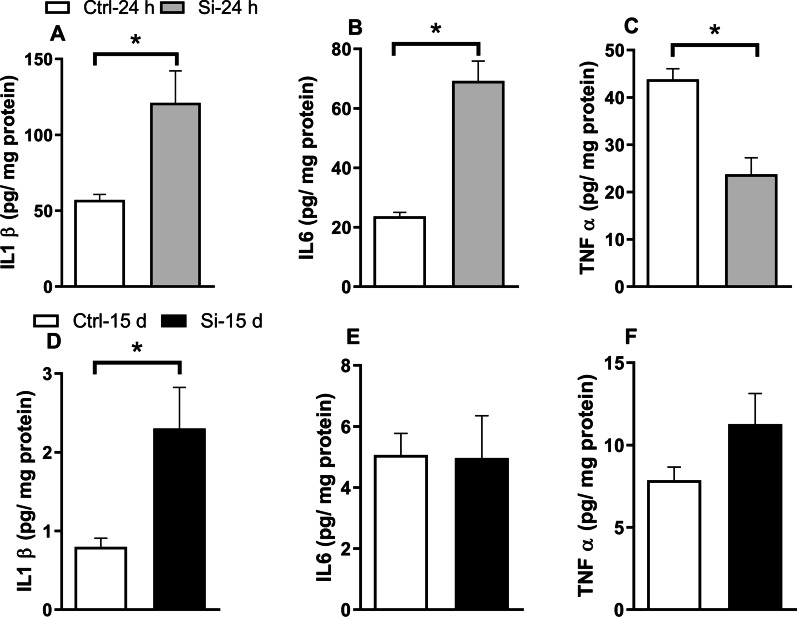
Lung pro-inflammatory cytokines in silica-instilled mice. Lung IL-1β, IL-6, and TNF-α were determined by ELISA 24 h (**A**–**C**; *N* = 8 Ctrl mice, 10 Si mice) or 15 d (**D**–**F**; *N* = 8 Ctrl mice, 9 Si mice) after intratracheal administration of saline (Ctrl) or silica (Si). Data are expressed as means ± SEM. *p < 0.05, Student’s *t*-test

Lung pro-inflammatory cytokines IL-1β [t (1,16) = 2.656; *p* = 0.0172] and IL-6 [t (1,16) = 5.997; *p* = 0.0001] were significantly increased 24 h after administration of silica (Fig. 2A, B). Cytokine levels were markedly reduced 15 days after administration of silica, compared to the levels found 24 h after instillation. Compared to Ctrl mice, lung IL-1β remained elevated 15 d after administration of silica [t (1,15) = 2.653; *p* = 0.0181], whereas IL-6 returned to baseline levels 15 days after instillation of silica (Fig. 2D, E). Intriguingly, lung TNF-α showed an initial decrease 24 h after administration of silica [t (1,16) = 4.529; *p* = 0.0003] followed by a trend of elevation at 15 days [t (1,15) = 1.605; *p* = 0.1294] (Fig. 2C, F).

### Intratracheal administration of silica induces hippocampal inflammation in mice

**Fig. 3:**
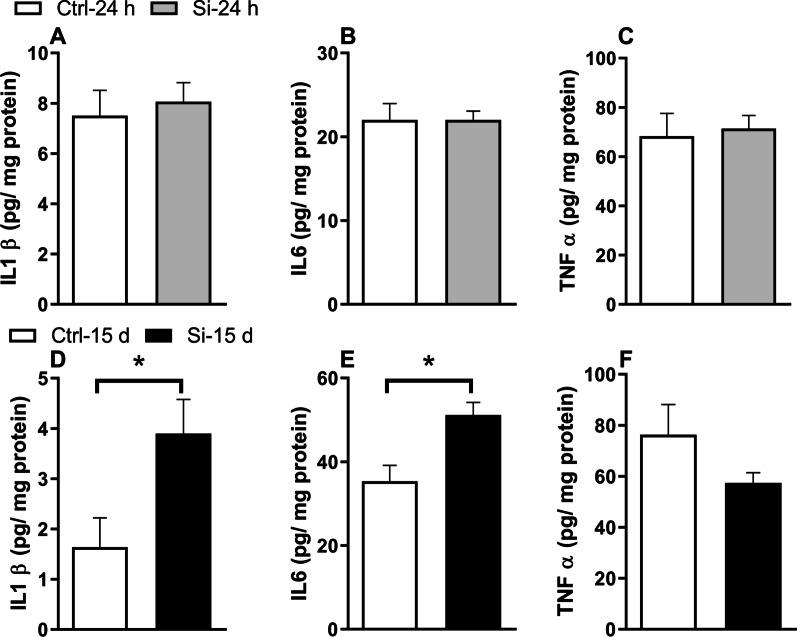
Hippocampal pro-inflammatory cytokines in silica-instilled mice. Hippocampal IL-1β, IL-6 and TNF-α were determined by ELISA 24 h (**A**–**C**; *N* = 8 Ctrl mice, 10 Si mice) or 15 d (**D**–**F**; *N* = 8 Ctrl mice, 9 Si mice) after intratracheal administration of saline (Ctrl) or silica (Si). Data are expressed as mean ± SEM. *p < 0.05, Student’s *t*-test

No differences in hippocampal pro-inflammatory cytokines (IL-1β, IL-6, TNF-α) were detected 24 h after intratracheal administration of silica in mice (Fig. 3A–C). Interestingly, however, hippocampal IL-1β [t (1,13) = 2.358; *p* = 0.0347], and IL-6 [t (1,15) = 3.543; *p* = 0.0030], but not TNF-α [t (1,15) = 1.668; *p* = 0.1175], were significantly increased (compared to Ctrl mice) 15 days after intratracheal administration of silica (Fig. 3D–F).

### Intratracheal administration of silica induces hippocampal synapse damage and increases Aβ42 levels in mice

**Fig. 4:**
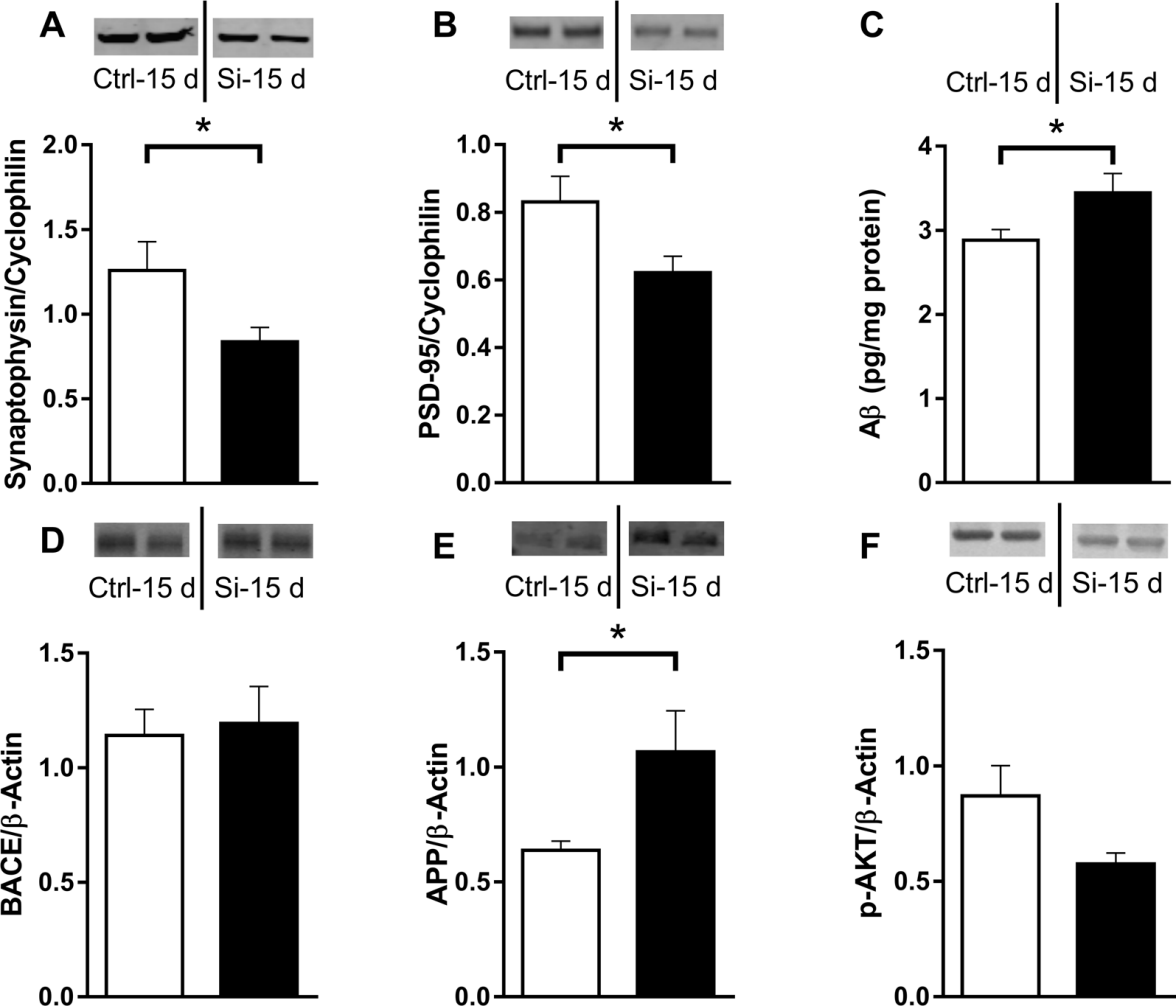
Intratracheal administration of silica induced hippocampal synapse damage and increased Aβ levels. Pre- and post-synaptic marker proteins (synaptophysin and PSD-95, respectively; **A**, **B**), Aβ42 (**C**), BACE1 (**D**), APP (**E**), and pSer473-Akt (**F**) were determined in hippocampal homogenates 15 days after intratracheal administration of silica (*N* = 9 Ctrl mice, 7 Si mice). Actin and cyclophilin were used as loading controls, as shown (bottom of the representative figures). Data are expressed as mean ± SEM. *p < 0.05, Student’s *t*-test

We next investigated whether the induction of pro-inflammatory cytokines induced by lung silicosis could entail synapse damage in the hippocampus. Pre- and post-synaptic markers, synaptophysin [t (1,14) = 2.310; *p* = 0.0413] and PSD-95 [t(1,10) = 2.326; *p* = 0.0423], respectively, were significantly reduced in hippocampal homogenates from silica-instilled mice (Fig. 4A, B).

The decreases in pre- and post-synaptic marker proteins in the hippocampi of silica-instilled mice were similar to previous observations in Alzheimer’s disease (AD) mouse models [31]. This led us to investigate if hippocampal inflammation in silica-instilled mice could be associated with changes in levels of the amyloid-β peptide (Aβ), a neurotoxin that accumulates in AD brains and is implicated in brain inflammation and synapse damage in AD [8, 30, 32–34]. Aβ was indeed elevated in the hippocampi of Si mice compared to Ctrl animals [t (1,14) = 2.157; *p* = 0.0488] (Fig. 4C).

To examine a possible mechanism underlying increased hippocampal Aβ in silica-instilled mice, we measured protein levels of β-secretase (BACE 1), a key protease involved in the cleavage of the amyloid precursor protein (APP) to release Aβ [35, 36]. No difference was observed in BACE1 immunoreactivity between Ctrl and Si mice [t (1,12) = 0.2663; *p* = 0.410] (Fig. 4D). However, APP was significantly increased in the hippocampus 15 days after administration of silica in mice [t (1,11) = 2.174; *p* = 0.05] (Fig. 4E), suggesting that the increase in Aβ might be related to increased substrate (APP) availability.

Recent studies have established that inflammation-associated inhibition of brain insulin signaling plays an important role in neurodegenerative mechanisms leading to synapse damage and cognitive impairments in AD [8] and sepsis [13, 37]. To determine whether a similar mechanism might be induced by silicosis-induced hippocampal inflammation, we examined pSer473-Akt levels as a proxy of activity of the insulin signaling pathway. pSer473-Akt was reduced in Si mice compared to Ctrl animals [t (1,12) = 2.124; *p* = 0.0551], indicating that brain inflammation induced by intratracheal administration of silica is accompanied by inhibition of hippocampal insulin signaling.

### Intratracheal administration of silica induces memory impairment in mice

**Fig. 5:**
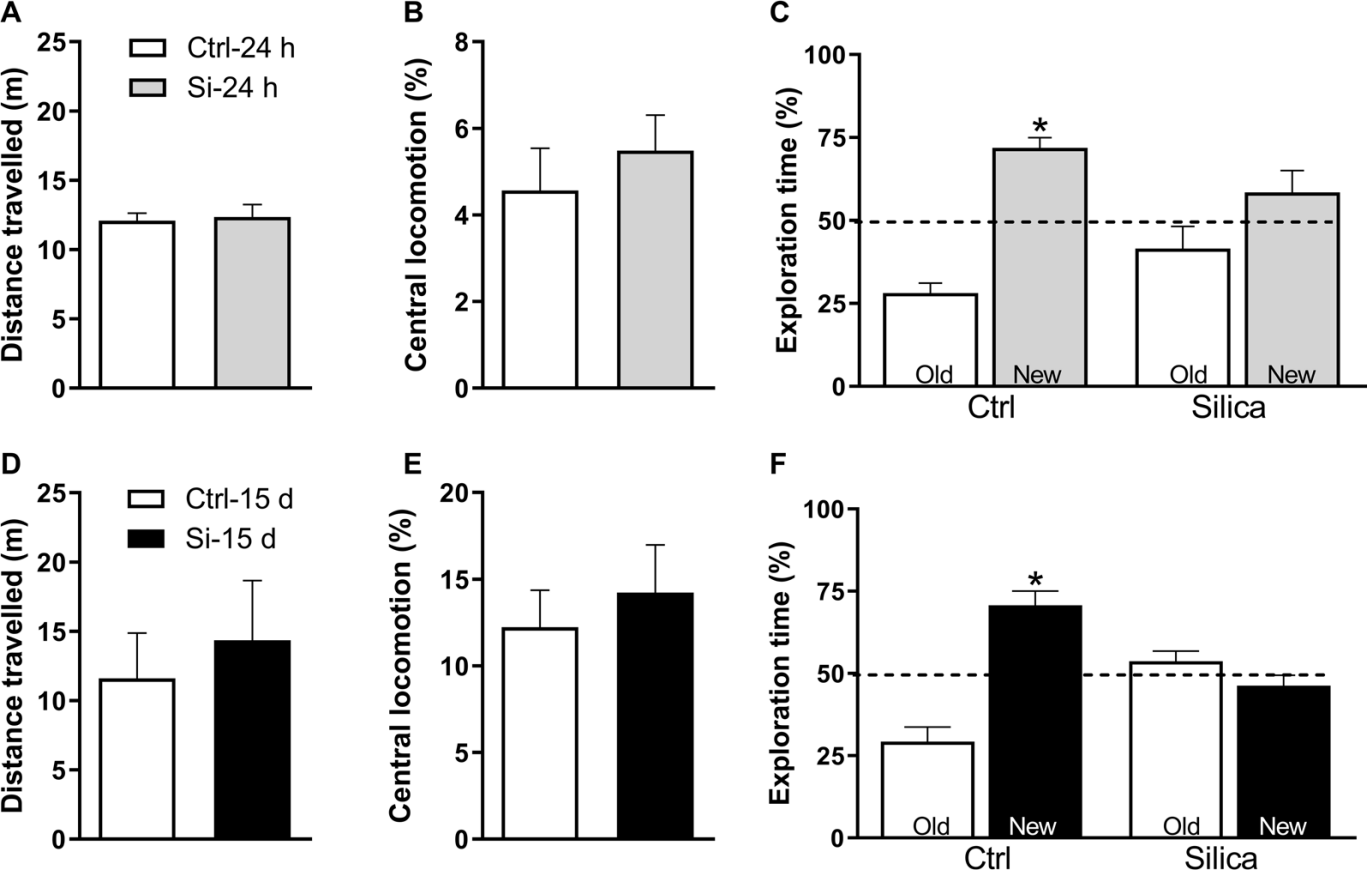
Intratracheal administration of silica induces memory impairment in mice. Mice were tested 24 h (**A–C**) or 15 d (**D–F**) after intratracheal administration of saline (Ctrl) or silica (Si). Open field tests showed no difference between groups in total distance traveled (**A** 24 h post-instillation, **D** 15 d post-instillation) or time spent at the center of the arena (**B** 24 h post-instillation, **E** 15 d post-instillation) (**A**, **B** N = 7 Ctrl mice, 8 Si mice; **D**, **E** N = 8 Ctrl mice, 9 Si mice). **C**, **F** Memory performance was assessed in the NOR paradigm 24 h (**C**; *N* = 7 Ctrl mice, 8 Si mice) or 15 d after administration of silica or saline (**F**; *N* = 8 Ctrl mice, 9 Si mice). Data are expressed as mean ± SEM. *p < 0.05, one-sample Student’s *t*-test compared to the fixed value of 50%. Old = familiar object; New = novel object

Finally, we hypothesized that the impact of silicosis on hippocampal pro-inflammatory cytokines, synaptic markers, and phosphorylation of Akt could result in memory impairments in mice. Control open field tests revealed no differences in total distance traveled or time spent at the center of the arena between Ctrl and Si mice, indicating that silica instillation did not affect locomotor/exploratory behavior or induced anxiety in mice (Fig. 5A, B, D, E).

As expected, Ctrl mice exhibited a clear preference for the novel object in the novel object recognition (NOR) test session, both 24 h [t (1,6) = 7.227; *p* = 0.0004] and 15 days [t (1,7) = 4.713; *p* = 0.0022] after saline administration. In contrast, silica-instilled mice failed to recognize the familiar object as such, and spent comparable amounts of time exploring familiar and novel objects, both 24 h [t (1,7) = 1.276; *p* = 0.2425] and 15 days [t (1,8) = 1.180; *p* = 0.2720] after administration of silica (Fig. 5C, F).

## Discussion

We initially established that a single dose of silica (2.5 mg/kg; particle size range: 500 nm–10 μm) administered into the trachea induced alveolar collapse, lung infiltration by PMN cells, and increased lung pro-inflammatory cytokines, all indicative of the induction of silicosis in mice. We further found that lung silicosis induced by this low dose of silica [38] was followed by an increase in hippocampal pro-inflammatory cytokines, synapse damage, accumulation of the Aβ peptide, and impaired memory.

In addition to an acute (24 h) examination of the impact of intratracheal administration of silica particles on the lung and brain, we analyzed lung and brain inflammation 15 days after administration of silica. The 15-day time window is frequently used to study the outcomes of exposure to SiO_2_ (e.g., Reiser et al. [39]; Faffe et al. [26]; Yang et al. [40]. Numerous studies have demonstrated significant pathological alterations induced by silica particles using a 15-day experimental window. The experimental design in our current study was thus based on a validated model in the silicosis field.

Silicosis is a restrictive disease that causes alveolar collapse, an increase in lung elastic tissue and lung infiltration by inflammatory cells [26]. The increase in collapsed alveoli indicates tissue damage and impairment of pulmonary function [41]. As a result, cytokines are secreted into the lung, triggering pulmonary remodeling processes, and resulting in the production of connective tissue fibers [41]. As expected, we found morphometric and cellularity alterations in the lung following intratracheal administration of silica. The percentage of collapsed alveoli was increased 24 h after instillation and remained elevated 15 days later, indicating that tissue damage was persistent over time. We further observed an increase in the number of PMN cells, but not in MN cells, in the lung 24 h after silica instillation (but not 15 days after instillation), indicating an acute lung infiltration by peripheral immune/inflammatory cells.

Previous studies employing administration of higher doses of silica (20 mg/kg) demonstrated that histological alterations in the lung were accompanied by robust increases in pro-inflammatory cytokines [42, 43]. Thus, we sought to determine whether the lower dose of silica employed here elicited a similar response. In accordance with previous results [3], both IL-1β and IL-6 were significantly increased in the lung 24 h after administration of silica. However, in contrast with the increase in lung TNF-α observed in mice instilled with a higher dose of silica [3], under our conditions TNF-α was reduced at 24 h in Si mice compared to Ctrl animals. The different TNF-α response may be related to the low dose of silica administered or to the use of Swiss mice in the current work, as opposed to a higher silica dose and BALB/c mice in previous studies [3]. In addition, evidence suggests that TNF-α release is time-dependent in silica-induced toxicity [44], and lipoxins may regulate this release as discussed next. In line with the reduction in PMN cell number, IL-6 and TNF-α retuned to baseline levels 15 days after silica instillation.

Modulation of cytokine levels in silicosis may be further be related to the upregulation of lipoxin A4 (LXA4) signaling. An increase in LXA4 has been found to contribute to the protective effect of apolipoprotein A1 (ApoA1) against fibrosis in an experimental model of lung silicosis [45]. The mechanism of LXA4 protection has been reported to involve attenuation of the release of pro-inflammatory cytokines and chemokines, such as TNF-α and macrophage inflammatory protein-2 [46]. In addition to its anti-inflammatory activity, LXA4 stimulates macrophages to perform phagocytosis of apoptotic immune cells without the release of pro-inflammatory cytokines [47].

Administration of micro- and nano-sized particulate matter to animals via the lung has been shown to trigger systemic inflammation and lesions to the spleen, heart, and kidney [40, 48], as well as autoimmunity [49]. Humans exposed to silica displayed increased levels of systemic inflammatory markers [50], rheumatoid arthritis or systemic scleroderma [51]. The central nervous system can also be impacted by silica. After inhalation, SiO_2_ nanoparticles penetrate the epithelium of the respiratory tract and are translocated to the brain via either the circulatory system or the olfactory nerve [52]. Following exposure to silica nanoparticles, mice showed neuropathology, degeneration, and synapse damage [3]. Silica nanoparticles were found to be primarily deposited in the frontal cortex and hippocampus [3]. Because the hippocampus is centrally implicated in memory and cognition, and pathological deregulation of hippocampal function underlies cognitive and memory impairments in neurological disorders, we investigated the possibility that lung inflammation might be followed by hippocampal inflammation and memory loss in silicosis.

Increasing evidence supports the notion that both acute and chronic peripheral inflammation can trigger brain inflammation, neurodegeneration, and cognitive deficits [2, 30, 37]. Significantly, recent studies have revealed that restrictive pulmonary diseases, such as silicosis, are risk factors for cognitive impairment and dementia [23]. However, the mechanisms underlying the association between lung and brain dysfunction remain unknown.

The potential crosstalk between lung inflammation and brain dysfunction in the mouse silicosis model was initially evaluated by measuring hippocampal cytokines. Results showed no significant differences in hippocampal IL-1β, IL-6, or TNF-α 24 h after administration of silica. However, hippocampal IL-1β and IL-6 were significantly increased 15 days after instillation, suggesting that hippocampus inflammation was triggered by and temporally followed the initial lung inflammation caused by silica.

Increased brain levels of pro-inflammatory cytokines trigger synapse damage and affect neuroplasticity [6], resulting in cognitive impairments [2, 32]. We found that hippocampal pre- and post-synaptic markers (synaptophysin and PSD-95, respectively) were significantly decreased 15 days after administration of silica, indicating synapse damage and loss. Numerous studies have established synapse loss as a hallmark of AD [33]. Soluble forms of the Aβ peptide accumulate in the AD brain [35, 36] and activate pro-inflammatory pathways leading to synapse loss and neuronal damage [7, 53]. This prompted us to investigate whether hippocampal inflammation in silica-instilled mice was associated with elevated Aβ levels. Hippocampal Aβ levels were indeed higher in silica-instilled than in control mice. While we did not detect any changes in BACE1, one of the secretases responsible for cleavage of APP and production of Aβ [54], APP was found to be elevated in the hippocampi of mice that received silica. It is, thus, possible that the increase in hippocampal Aβ resulted from increased substrate (APP) availability for cleavage by secretases in silica-instilled mice. Results suggest that inflammatory signaling from the lung triggers a deleterious process involving elevated hippocampal cytokines and Aβ and leading to synapse damage in the hippocampus. Communication via the vagus nerve might be an additional mechanism involved in the crosstalk between peripheral and central inflammation in silicosis, and further studies appear warranted to investigate this communication.

Inhibition of brain insulin signaling is thought to play a major role in the pathogenesis of neurodegenerative disorders [7, 8, 37] and in sepsis [24, 25], and to be a leading mechanism underlying cognitive impairment. A key component of the insulin signaling pathway is Akt, which becomes phosphorylated at Ser473 in response to insulin [8]. Disruption of Akt signaling is detrimental to insulin response and may lead to brain dysfunction [7]. Our results showed a decrease in hippocampal pSer473-Akt in silica-instilled mice, suggesting disruption of insulin signaling such as observed in AD and other neurodegenerative disorders.

Finally, our findings of hippocampal inflammation, synapse damage and impaired insulin signaling prompted us to assess the impact of the administration of silica on memory. Remarkably, we found that intratracheal instillation of a low-dose of silica impaired memory in the NOR test.

**Fig. 6:**
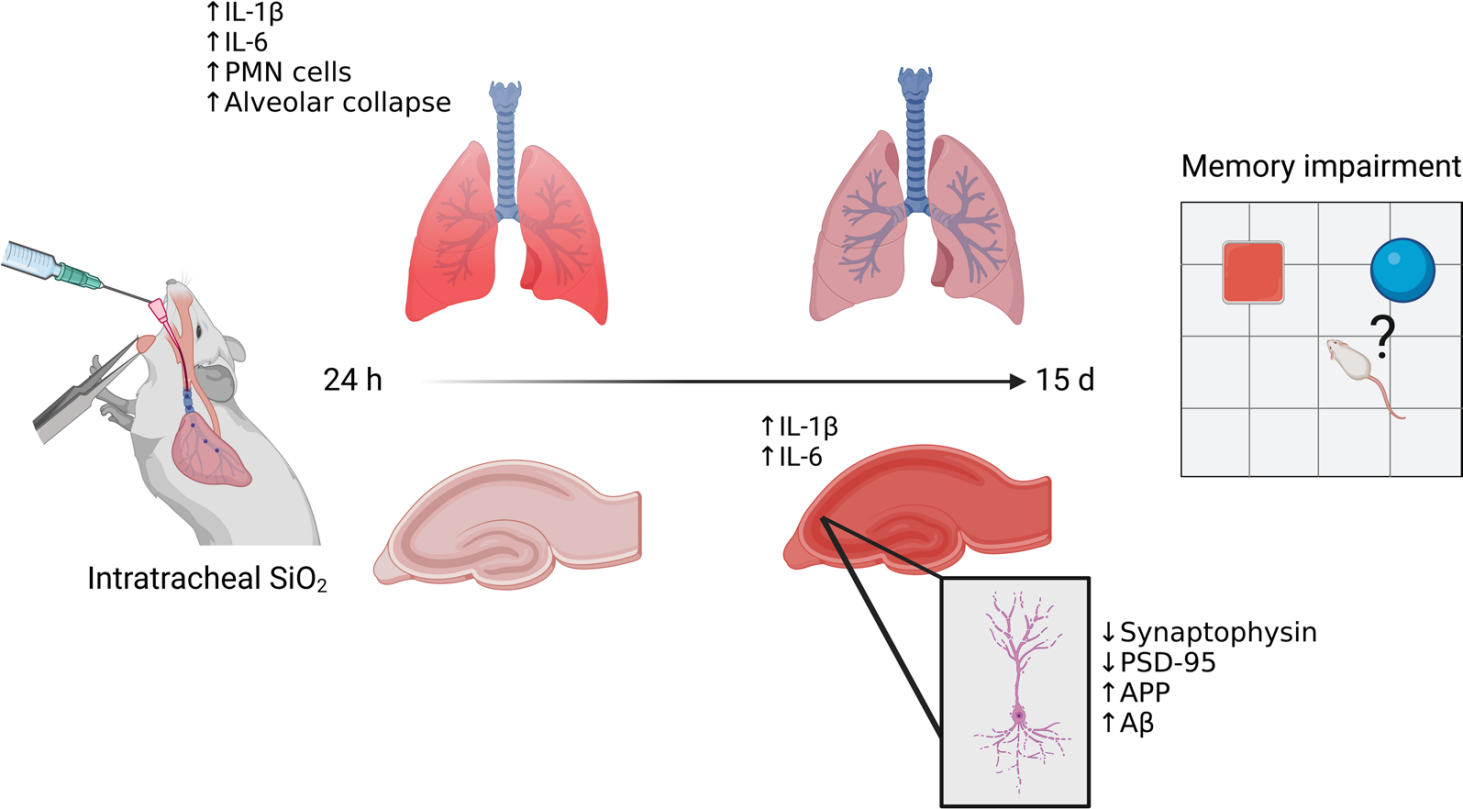
Schematic representation of the current study findings. Hippocampal inflammation temporally follows the initial lung inflammation induced by silica particles. Cytokine (IL-1β and IL-6) levels first increase in the lung 24 h after intratracheal administration of silica, coinciding with lung infiltration by phagocytes (PMNs). The initial lung inflammatory response is followed by increased hippocampal IL-1β and IL-6 15 d after silica instillation. This, in turn, is accompanied by damage to synapses, leading to memory impairments

In conclusion, the current study identified a crosstalk between lung and brain leading to hippocampal inflammation, synapse damage and cognitive impairment after a single intratracheal instillation of a low dose of silica in mice. Figure 6 schematically represent this sequence of events. Interestingly, a recent study showed a link between intranasal exposure to silica, α-synuclein aggregation and neurodegeneration in a Parkinson’s disease model [55]. In light of recent clinical and epidemiological studies connecting pulmonary dysfunction with cognitive impairments and dementia [22–25], and considering the prevalence of silicosis as an occupational disease, investigation of potential neurological outcomes in patients appears warranted. Our findings further suggest that early intervention to attenuate peripheral pro-inflammatory signaling may be important to preserve brain health in silicosis.

## Data Availability

The dataset(s) supporting the conclusions of this article are available in the OSF repository on https://osf.io/rp4kq/.

## Abbreviations

AD: Alzheimer’s disease
APP: Amyloid precursor protein
Aβ: Amyloid-β peptide
IL-1β: Interleukin-1β
IL-6: Interleukin-6
i.t.: Intratracheal
LXA4: Lipoxin A4
MN: Mononuclear cells
NOR: Novel object recognition test
PMN: Polymorphonuclear cells
TLRs: Toll-like receptors
TNF-α: Tumor necrosis factor-α
BACE 1: β-Secretase
